# A systematic review and meta-analysis of systematic and topical tranexamic acid administration in aesthetic plastic surgery

**DOI:** 10.1186/s13741-024-00406-7

**Published:** 2024-06-03

**Authors:** Jerzy Kolasiński, Tomasz Reysner, Małgorzata Kolenda, Szymon Kołacz, Małgorzata Domagalska

**Affiliations:** 1 Kolasinski Clinic, Hair Clinic Poznan, 62-020 Swarzędz, Poland; 2 Department of Palliative Medicine, Poznan University of Medical Sciences, 61-245 Poznań, Poland

## Abstract

**Introduction:**

Tranexamic acid has been widely used in plastic surgery. However, its efficacy has yet to be fully established. This meta-analysis aimed to determine its effectiveness in aesthetic plastic surgery.

**Methods:**

Following PRISMA guidelines, we conducted a meta-analysis of prospective randomised clinical trials that compared the effects of topical or systematic administration of tranexamic acid versus the control group in aesthetic plastic surgeries. The study was registered on the International Register of Systematic Reviews (PROSPERO) and is available online (www.crd.york.uk/prospero, CRD42023492585).

**Results:**

Eleven studies encompassing 960 patients were included for the synthesis after critical evaluation. Systematic (*MD* − 18.05, 95% *Cl*, − 22.01, − 14.09, *p* < 0.00001) and topical (*MD* − 74.93, 95% *Cl*, − 88.79, − 61.07, *p* < 0.00001) administration of tranexamic acid reduced total blood loss. Topical tranexamic acid reduced drainage output (*p* < 0.0006).

**Conclusion:**

Tranexamic acid reduced blood loss in aesthetic plastic surgery. More strictly defined RCTs, using high-quality methodology, are needed to evaluate the advantages and disadvantages of tranexamic acid in aesthetic plastic surgery.

## Introduction

Although most plastic surgery procedures do not cause significant blood loss, bleeding can cause swelling, bruising, and pain, require fluid and dressing changes and repeat procedures, and increase the risk of voluntary infection and wound rapture. Therefore, surgical blood collection has minimal benefit to the patient and the doctor. Fear of bleeding may also prevent plastic surgeons from using adequate deep-vein thrombosis prophylaxis (Badireddy and Mudipalli 2018).

The balance between coagulation and fibrinolysis must be maintained to prevent bleeding and maintain circulation during surgery. If fibrinolysis exceeds coagulation, bleeding may occur despite adequate haemostasis. While careful surgical techniques in soft tissue surgery, cautery, and local epinephrine infiltration of the wound can control bleeding, bone cannot be penetrated or used for the same. Therefore, measures such as hypotensive anaesthesia and drug support are used to prevent bone bleeding.

The term “plastic surgery” covers a wide range of procedures. Medical and cosmetic uses of tranexamic acid have been explored in plastic surgery, mainly involving skeletal structures such as rhinoplasty, craniomaxillofacial, and orthognathic surgery. Aesthetic surgery mainly involves procedures that improve the appearance of the face and body. Cosmetic surgery offers help for people who feel self-conscious or unhappy with their appearance. This includes tummy tuck, breast augmentation, eyelid surgery, and liposuction. Most aesthetic plastic surgery occurs in a tissue, and tissue-dependent effects of tranexamic acid may be as significant as those in specific surgery.

Antifibrinolytic drugs are generally available only by pharmacological means, which reduce bleeding and have a stable profile in patients without coagulation deficiencies (Shah et al. 2020). Tranexamic acid (TXA) is a synthetic, low-cost antifibrinolytic drug developed and approved for intravenous and oral use. This anticoagulant drug can reduce blood loss and the need for blood transfusion (Levack et al. 2020; Jejani et al. 2020; Shi et al. 2022).

TXA is a synthetic lysine analogue that prevents the breakdown of fibrin clots. It is considered a reasonable precaution because it is effective in many surgeries (Patel et al. 2022). It was initially used to treat bleeding disorders, oral bleeding, and epistaxis (Cai et al. 2020). TXA is now widely used in elective and emergency surgery, but its indications are still debated. TXA can cause complications such as heart disease and epilepsy. However, it is a clinical practice, and most studies show it is a safe and effective antifibrinolytic drug (Zak et al. 2021; Madathil et al. 2021). TXA is effective when administered systematically and topical. However, the ideal method of administration has not been established (Parmeshwar et al. 2023; Xu et al. 2021). Its oral bioavailability is 30 to 50%, and its renal elimination is more significant than 95%. After intravenous injection, plasma concentrations rapidly reach a high level. Its half-life in adults is approximately 3 h. However, it remains in tissues for up to 17 h (Grassin-Delyle et al. 2022). The safety and effectiveness of TXA have been well-studied in cardiology (Shi et al. 2022; Madathil et al. 2021), orthopaedics (Groene et al. 2021; Wong et al. 2021; Bloom et al. 2023), and other specialities (Tsan et al. 2023; Mocanu et al. 2023; Simsam et al. 2023). Also, TXA reduces the likelihood of blood transfusion by one-third and reduces the transfused blood by one unit (Patel et al. 2022). TXA reduces the risk of death in trauma patients also after brain injuries.

TXA is rarely used in aesthetic plastic surgery, except in craniofacial procedures. However, since the last review by (Laikhter et al. 2022), the number of articles examining the use of TXA in aesthetic plastic surgery has increased. The previous review evaluated the risk of bias only with the Cochrane risk-of-bias tool, and the GRADE evidence score was not assessed. This systematic review and meta-analysis aimed to update readers on the current knowledge and clinical recommendations regarding the efficacy of TXA in aesthetic plastic surgery based on good-quality evidence.

## Methods

This systematic review and meta-analysis tracked the instructions of the Preferred Reporting Items of Systemic Reviews and Meta-Analyses (PRISMA) guidelines. The International Register of Systematic Reviews (PROSPERO) certified the study protocol and is accessible online (www.crd.york.uk/prospero, CRD42023492585). The focused question was as follows: Does TXA reduce blood loss during aesthetic plastic surgery? Does TXA affect the drainage output after aesthetic plastic surgical procedures? Therefore, the PICO question was as follows: for adults undergoing aesthetic plastic surgery (P), does the TXA (I) compared to placebo or no intervention (C) lower blood loss or drainage output?

### Search strategy and quality assessment

The research strategy was conducted using clinical topics, and the main themes were based on the target population, intervention, control, and research question described previously. Two authors reviewed the literature through four electronic databases: PubMed, Google Scholar, MEDLINE, and Embase from the inception to January 2023. The Google Scholar search was restricted to the first 200 records. We evaluated studies using the following search terms: “tranexamic acid” (title), “abdominoplasty” (title), “plastic surgery”, “aesthetic surgery” (title), “rhinoplasty” (title), “liposuction” (title), “mastopexy” (title), “rhytidectomy” (title), and “breast augmentation” (title). Within each concept, authors combined the controlled words (Medical Subject Headings terms) and free keywords with the Boolean operators OR and AND. The titles, abstracts, and full texts of published studies were screened. Also, the reference lists of picked manuscripts were checked to prevent omitting any worthy trials. There was no limitation concerning the publication language.

### Eligibility criteria

#### Inclusion criteria

We included published journal manuscripts that met the following criteria:

- Adult or paediatric patients undergoing plastic surgical procedures who received TXA as an intervention
- All routes of TXA administration
- Placebo as a comparator
- Reported at least one of the outcomes: total blood loss, haemoglobin levels, drainage output, and hematoma
- Study design as an RCT
- Only full-text manuscript

#### Exclusion criteria

- Animal and in vitro studies
- Conference abstracts, reviews, letters, case reports, or editorials and non-peer-reviewed sources
- No-RCT studies
- Studies with bleeding effects not assessed
- Studies with a high risk of bias or poor methodological quality
- No full-text manuscripts

Two authors comprehensively defer article inclusion, with all controversy inspected for final admittance by the senior author. As a result, this meta-analysis incorporated only randomised clinical trials.

### Data extraction and outcome assessment

Two reviewers autonomously extracted essential variables from the enclosed studies. The senior author revised any discrepancies during information extraction. Outcomes from the enclosed articles have been outlined as a meta-analysis to recognise established aspects in this literature, as seen in Table 1.

**Table 1:** Studies included in the meta-analysis

Year	Author	Jadad score	Type of study	Sample size	Application of TXA	Dose of TXA	Methods	Results
**Breast surgery**								
2023	Safran et al. (Safran et al. 2023)	5	RCT	82	Topical	3.0 g	TXA vs. placebo	Decrease in drain output in mastectomy
2023	Yao et al. (Yao et al. 2023)	5	RCT	98	Topical	1.0 g	TXA vs. placebo	TXA did not decrease the incidence of haematoma and in drainage output
2020	Ausen et al. (Ausen et al. 2020)	5	RCT	202	Topical	0.5 g	TXA vs. placebo	TXA reduce the blood loss
**Liposuction**								
2023	El Minawi et al. (Minawi et al. 2023)	5	RCT	90	Topical and systematic	8 mg/kg	TXA topical vs. TXA systemic vs. placebo	TXA reduced the blood loss
2022	Hayo et al. (Hoyos et al. 2022) ha	5	RCT	141	Topical and systematic	1.0 g	TXA topical vs. TXA systemic vs. placebo	TXA groups had higher haemoglobin levels
**Rhinoplasty**								
2017	Ghavimi et al. (Ghavimi et al. 2017)	5	RCT	50	Systemic	10 mg/kg	TXA vs. placebo	Decrease intraoperative bleeding
2016	Eftekharian et al. (Eftekharian and Rajabzadeh 2016)	5	RCT	50	Systemic	1.0 g	TXA vs placebo	Decrease intraoperative bleeding
2015	Beikaei et al. (Beikaei et al. 2015)	5	RCT	100	Systemic	10 mg/kg	TXA vs placebo	Decrease intraoperative bleeding
2022	Habibi et al. (Habibi et al. 2022)	5	RCT	198	Topical	5 mg/kg	TXA vs placebo	Decrease intraoperative bleeding
2021	Avci et al. (Avci 2021)	5	RCT	90	Systemic	1 g	TXA vs placebo	Decrease intraoperative bleeding

Mean and standardised deviation (SD) were directed to describe the extracted data. When median and range or interquartile range (IQR) were inclined, the mean and SD were approximated using the Cochrane RevMan Web calculator tool. For studies that declared no or unsatisfactory data, we attempted to contact authors to receive the missing data. When possible, data were inferred from tables or figures. The software GetData Graph Digitizer (v2.26, Canopus, Japan) quoted the outcome values when the aftermath was reported as a chart.

### Risk-of-bias assessment

Two authors separately calculated the included trials to determine their methodological features. We used the Cochrane risk-of-bias tool and the Jadad scale for randomised studies.

**Fig. 1:**
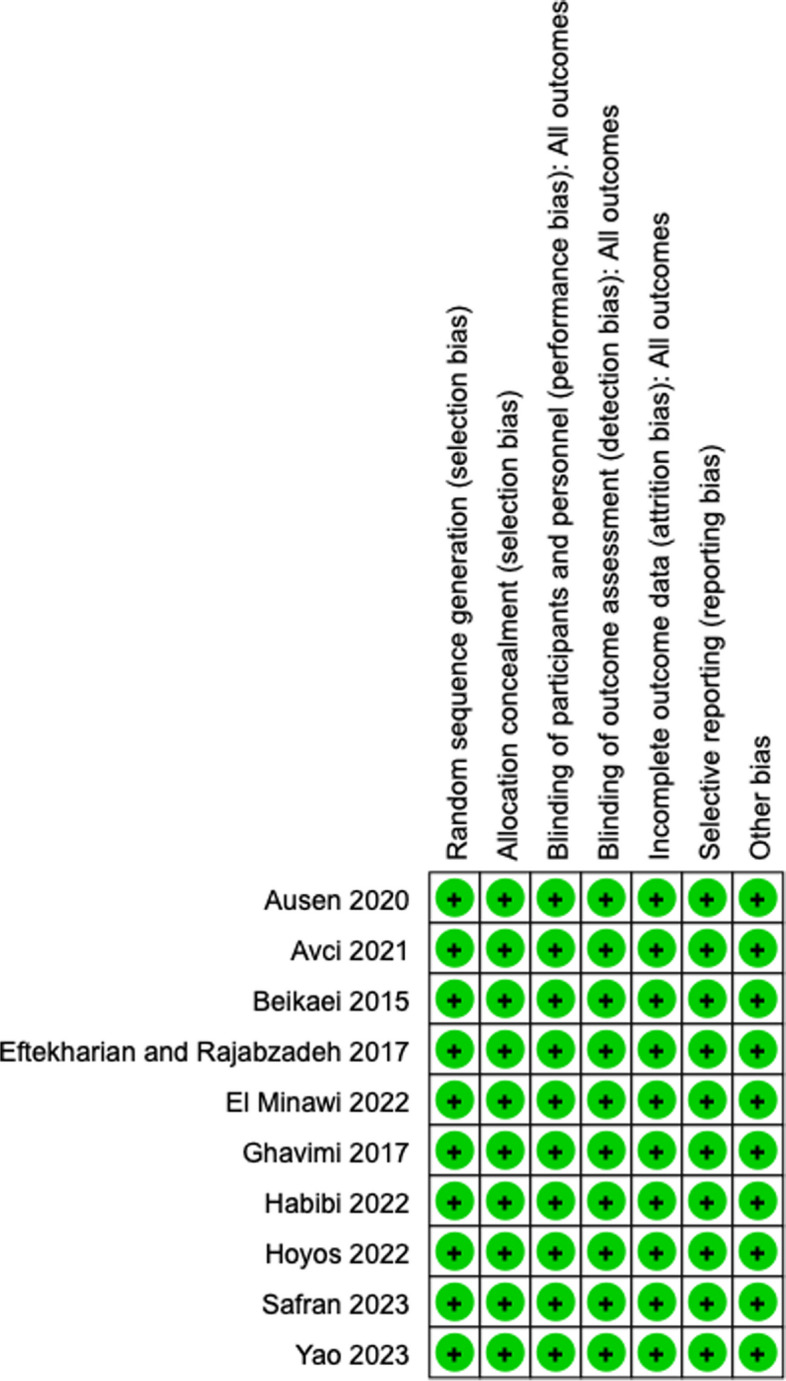
Risk of bias

The checklist contained questions on randomisation (selection bias), deviations from the intended interventions (performance bias), measurement of the outcome (detection bias), selection of the reported result (reporting bias), and overall bias. In cases of disagreement between the examiners, a third reviewer was consulted. The risk of bias in the chosen studies was evaluated as low risk, unclear risk of bias, or high risk of bias. The Cochrane risk of bias of the tabbed studies is presented in Fig. 1.

In the Jadad scale, the questions included the following: Was the study described as randomised? Was the study defined as double-blinded? And was there a description of withdrawals and dropouts? Each question was to be answered yes or no. Each yes scored a single point. Additional points were also given if the randomisation method was described and the methodology was appropriate or if the blinding process was described and relevant. An RCT could receive a Jadad score of between 0 and 5. The Jadad scores of the selected studies are shown in Table 1.

### Data synthesis and analysis

Meta-analysis was performed using Cochrane RevMan Web. The mean difference (MD) and 95% confidence interval (CI) were summarised statistics. A random-effects method was performed for meta-analysis. The level of significance was agreed at 0.05. *I*^2^ and chi-square tests were accomplished to qualify the heterogeneity across studies.

After the meta-analysis of every admitted outcome, the quality of evidence was calculated with the Grading of Recommendations Assessment, Development and Evaluation (GRADE) system (Gopalakrishna et al. 2014). Based on assessment results in five aspects (risk of bias, inconsistency, indirectness, imprecision, and public bias), evidence was graded as very low, low, moderate, or high. A *p* < 0.05 was considered statistically significant.

## Results

### Study selection

**Fig. 2:**
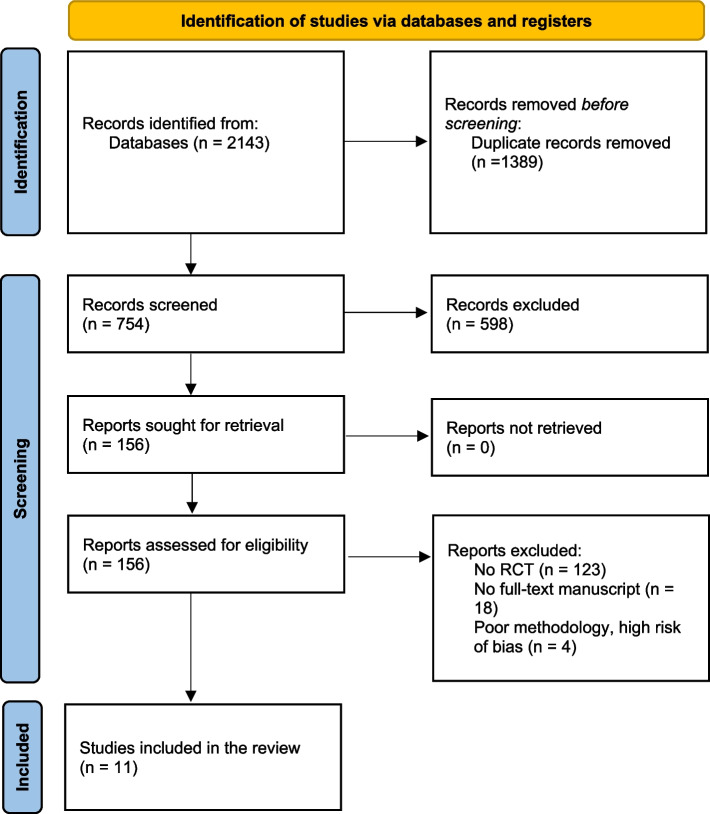
PRISMA flow diagram

The primary search yielded 2143 articles. Subsequently, 1389 duplicates were removed, the article titles and abstracts were viewed, and 598 irrelevant articles were excluded. One hundred forty-five articles were excluded due to failure to meet the inclusion criteria. Eleven relevant articles were picked based on relevance, citations, search quality, and recentness. The entire process is outlined in Fig. 2.

### Characteristics of included studies

Table 1 summarises the included randomised clinical trials. Three studies concerned breast surgery, two studies concerned liposuction, and five studies treated rhinoplasty. Four studies applied topical TXA administration. Three studies applied systemic TXA administration. Two studies applied systemic and topical administration of TXA. The dose of TXA varied from 0.5 g to 10 mg/kg. The sample size of the studies ranged from 50 to 202 patients.

### Total blood loss

#### Systematic TXA

**Fig. 3:**
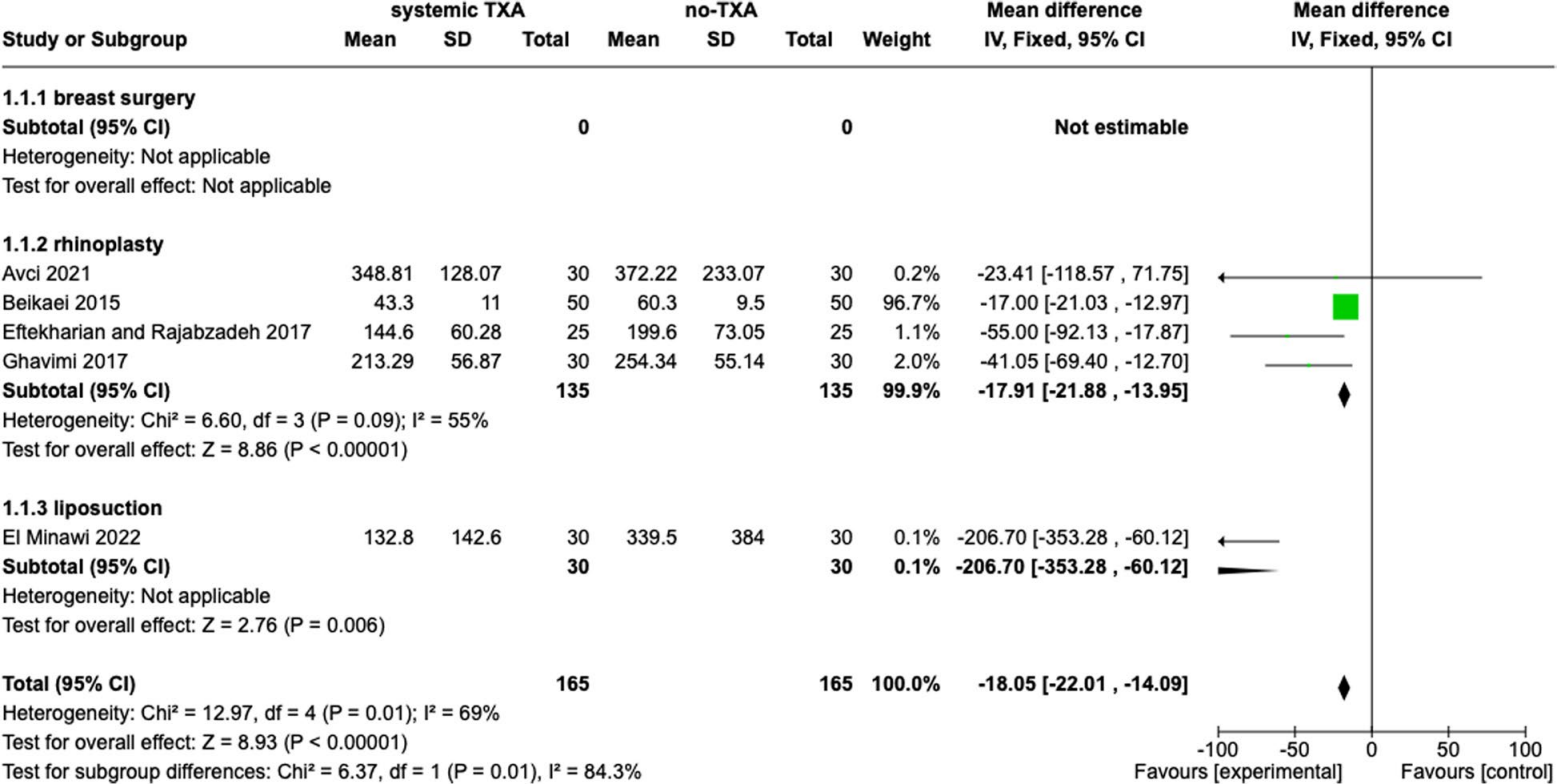
Total blood loss, systematic tranexamic acid

Five studies evaluated total blood loss. The data were accessible in all five studies, including 330 patients. The total blood loss was defined by the millilitres of the maximum blood loss after the surgery. The combination data showed a significant difference between the TXA group and placebo (− 18.05, 95% *Cl*, − 22.01, − 14.09, *p* < 0.00001, *I*^2^ = 69%). The subgroup analysis confirmed these findings. The results are presented in Fig. 3.

#### Topical TXA

**Fig. 4:**
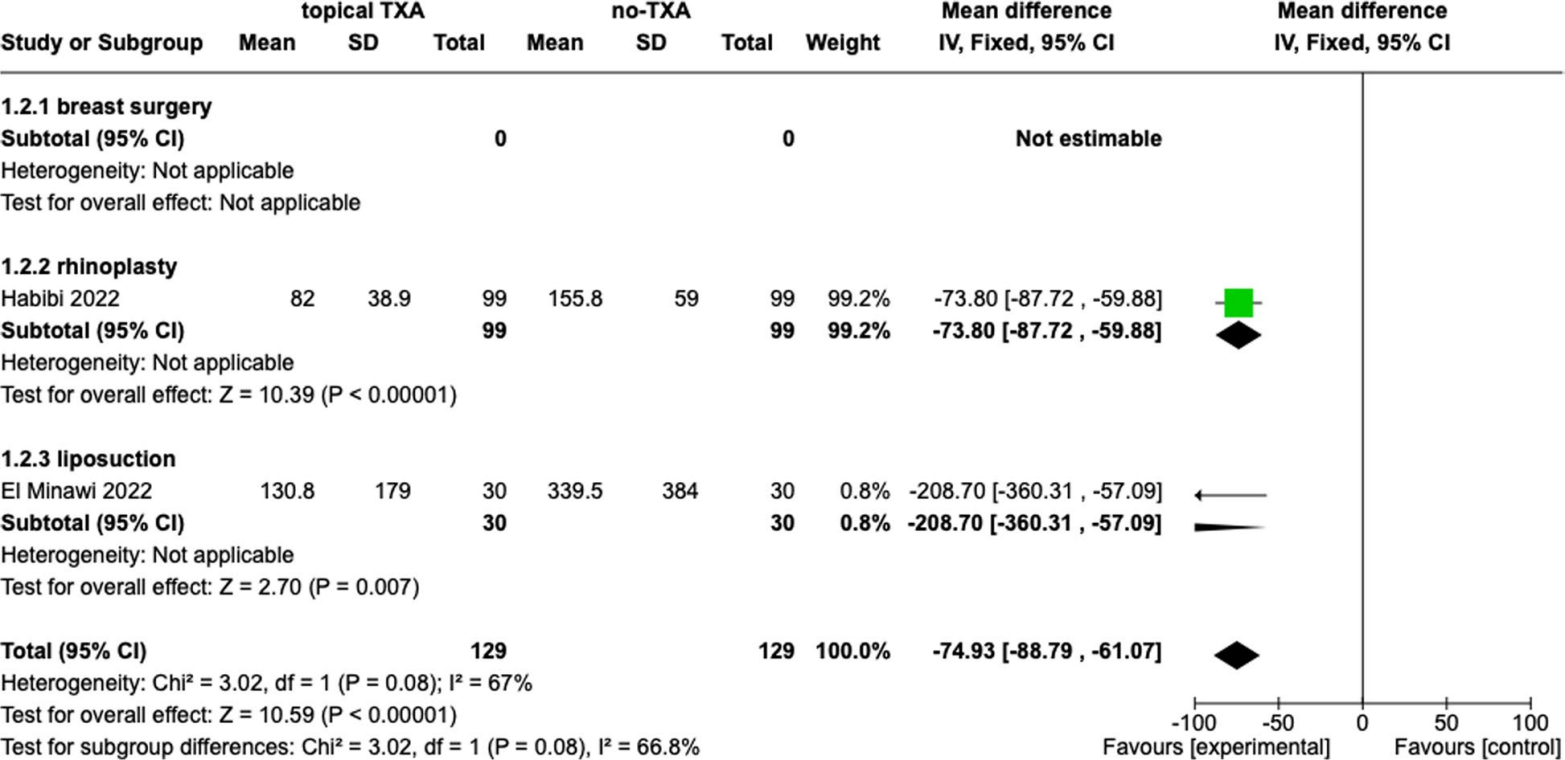
Total blood loss, topical tranexamic acid

Two studies evaluated total blood loss. The data were accessible in all two studies, including 258 patients. The total blood loss was defined by the millilitres of the maximum blood loss after the surgery. The combination data showed a significant difference between the TXA group and placebo (− 74.93, 95% *Cl*, − 88.79, − 61.07, *p* < 0.00001, *I*^2^ = 67%). The subgroup analysis confirmed these findings. The results are presented in Fig. 4.

### Drainage output

#### Topical TXA

**Fig. 5:**
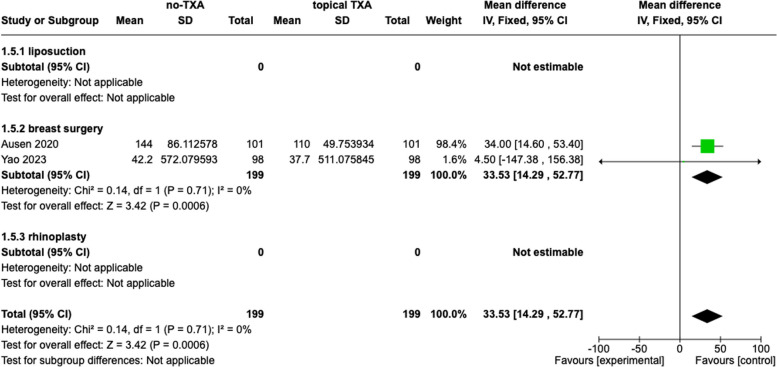
Drainage output, topical tranexamic acid

Two studies evaluated drainage output. The data were accessible in all two studies, including 398 patients. The drainage output was defined by the millilitres of the maximum drainage output after the surgery. The combination data showed a significant difference between the TXA group and placebo (33.53, 95% *Cl*, 14.29, 52.77, *p* < 0.0006, *I*^2^ = 0%). The subgroup analysis confirmed these findings. The results are presented in Fig. 5.

### Haematoma

**Fig. 6:**
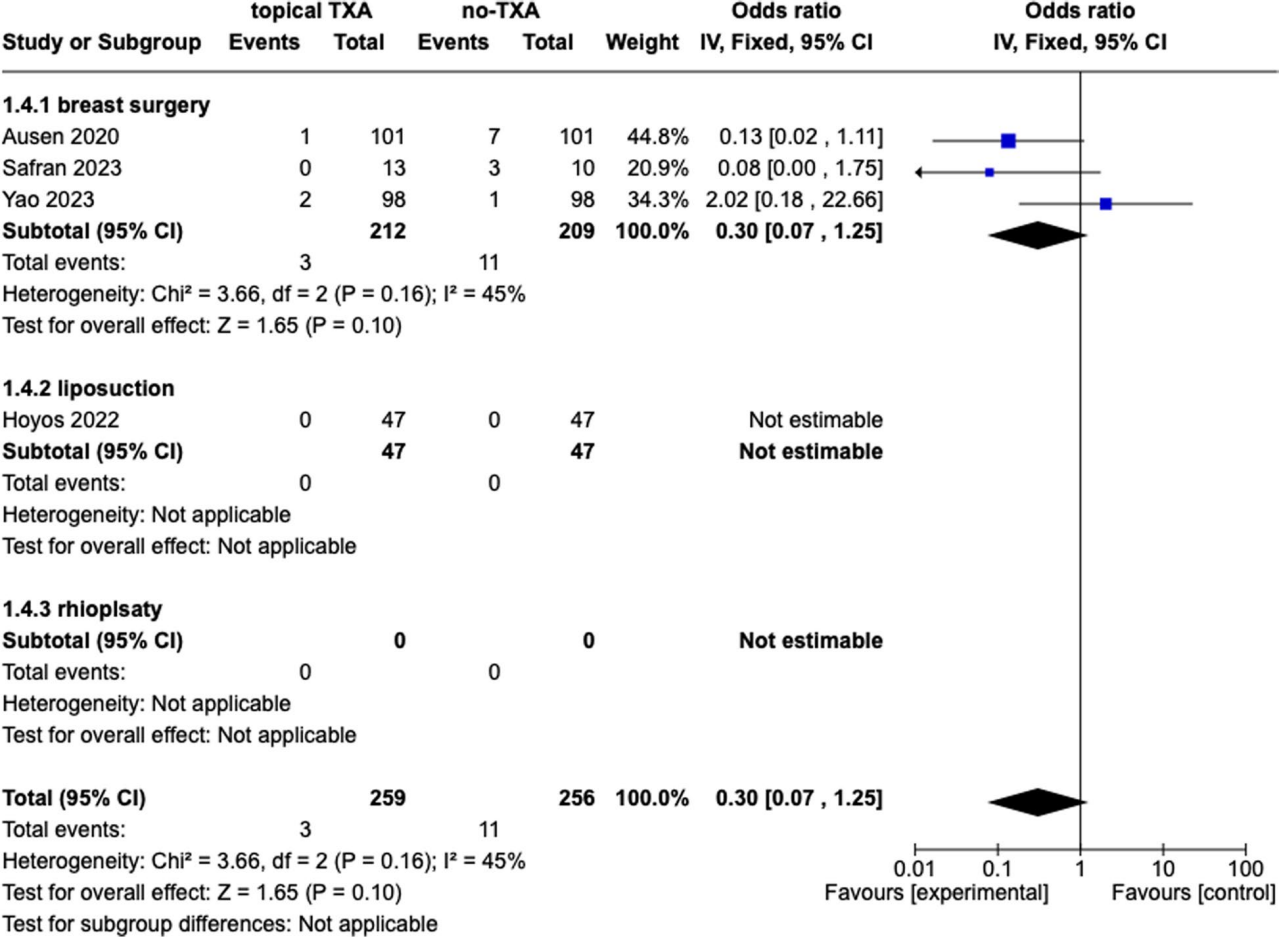
Haematoma, topical tranexamic acid

Two studies reported the presence or absence of postoperative haematoma. The data were accessible in all two studies, including 515 patients. The combination data showed a significant difference between the TXA group and placebo (33.53, 95% *Cl*, 14.29, 52.77, *p* < 0.0006, *I*^2^ = 0%). Overall, 11 patients who received a placebo and 3 patients who received TXA experienced postoperative haematoma. The subgroup analysis confirmed these findings. The results are presented in Fig. 6.

### GRADE evidence

Table 2 shows the analysis regarding the GRADE evidence judgement. The level of evidence was moderate for the total blood loss and for the systemic and topical TXA. The level of evidence was high for the hematoma and drainage output.

**Table 2LE: Table 3 has been renumbered to Table 2 including its citation. Please check if the action taken is appropriate and amend if necessary.** The GRADE evidence

No. of studies	Study design	Quality assessment					No. of patients		Effect	Quality
		Risk of bias	Inconsistency	Indirectness	Imprecision	Other	Adjuvant	Control	Absolute (95% *Cl*)	
Total blood loss — systemic TXA										
5	RCT	Not serious	Serious^a^	Not serious	Not serious	No	165	165	*MD* − 18.05 (− 22.01; − 14.09)	+ + + Moderate
Total blood loss — topical TXA										
2	RCT	Not serious	Serious^b^	Not serious	Not serious	No	129	129	*MD* − 74.93 (− 88.79; − 61.07)	+ + + Moderate
Drainage output — topical TXA										
2	RCT	Not serious	Not serious	Not serious	Not serious	No	199	199	*MD* 33.53 (14.29; 52.77)	+ + + + High
Haematoma –– topical TXA										
4	RCT	Not serious	Not serious	Not serious	Not serious	No	259	256	*MD* 0.30 (0.07, 1.25)	+ + + + High

*RCT* randomised controlled trial, *Cl* confidence interval, *PNB* peripheral nerve block, *MD* mean difference. ^a^Heterogeneity: *I*^2^ = 69%. ^b^Heterogeneity: *I*^2^ = 67%

## Discussion

The presented systematic review and meta-analysis included 10 RCTs to evaluate the efficacy of TXA in aesthetic plastic surgery. Pooled results of the available data showed that TXA significantly lowers total blood loss compared to placebo, regardless of the route of administration.

The growing number of studies investigating the use of TXA in cosmetic plastic surgery suggests an opportunity to expand the use of this drug in the aesthetic speciality. Originally developed as an antidote to prevent excessive bleeding and haemorrhage, the use of TXA in cosmetic procedures aims to achieve different results that are important for this group of patients. Previous reviews evaluating TXA concluded it effectively reduced bleeding, mainly in the cranial vault and orthognathic surgery (Wang et al. 2023). The reviews concerning aesthetic surgery also showed reduced bleeding, eyelid oedema, and ecchymosis, but they did not evaluate the Jadad scale and GRADE evidence (Laikhter et al. 2022; Locketz et al. 2020).

This systematic review and meta-analysis summarises bleeding outcomes from RCTs with a low risk of bias and good methodological quality, evaluating TXA in aesthetic plastic surgery using evidence-based methods.

Tranexamic acid is a synthetic lysine analogue developed by S. Okamoto in 1957 and is currently classified as an essential medicine by the World Health Organization (WHO). Although it has been approved by the US Food and Drug Administration (FDA) for the treatment of haemophilia, studies are ongoing investigations of its prescription for other medical uses. Despite its use in medicine for over 60 years, research on TXA continues to provide updates on its applications.

The application of TXA for hemostatic purposes in orthopaedic (Groene et al. 2021; Wong et al. 2021; Goldstein et al. 2022), oncological (Fowler et al. 2022; Liechti et al. 2022), and cardiac surgery (Houston et al. 2020; Chunmei et al. 2022) is well documented. However, the use of TXA in aesthetic plastic surgery remains controversial due to the scarcity and quality of the studies and the wide range of aesthetic plastic surgery procedures. Therefore, this meta-analysis may provide more reliable evidence supporting the application of TXA during aesthetic plastic surgeries. Our meta-analysis represents the first study to review only aesthetic plastic surgery with a low risk of bias or good methodological quality.

Based on 10 randomised controlled studies, this review found that the group receiving TXA had significantly better results than the control group. We proved that TXA reduces total blood loss, drainage output, and haematoma incidence compared to the placebo group.

However, in our systematic review and meta-analysis, significant heterogeneity was observed in the blood loss volume analysis, mainly due to differences in surgery type, blood loss volume calculation method (Gerdessen et al. 2021; Sokoliuk and Levchenko 2022), and TXA dose (Taam et al. 2020; Lam et al. 2023). There may be significant differences in blood loss volume between different surgeries, such as breast aesthetic surgery, liposuction, or rhinoplasty. Studies include measuring blood loss by calculating blood volume, changes in the number/weight of surgical sponges, or calculating blood loss volume using appropriate standards. According to a meta-analysis by Gerdesssen et al., techniques have a significant advantage in real-time blood loss assessment. Unfortunately, colourimetric techniques are rarely used in plastic surgery. None of the studies included in this meta-analysis uses calorimetric methods to evaluate blood loss. All of the studies included in this meta-analysis use direct measurement of blood loss.

Another reason for the significant heterogeneity of the blood loss results is that different doses of the TXA were used across the included studies. The doses of the intravenous TXA differ from 500 mg to 1.0 g. There is no consensus on the recommended dose of TXA in the plastic surgery. Heyns et al. (2021) showed that the most frequently used surgery dose was 15 mg/kg TXA with no incidence of venous thromboembolic events. In the studies included in our meta-analysis, the most commonly used dose of TXA was 10 mg/kg or 1.0 g.

However, our results regarding blood loss are similar to those of other meta-analyses regarding plastic aesthetic (Laikhter et al. 2022; Locketz et al. 2020) and non-plastic surgeries (Koh et al. 2022; Masouros et al. 2022).

Although fluid removal techniques often vary between hospitals, fluid drainage is one way to reduce the incidence of seromas and discomfort associated with fluid replacement (Hui et al. 2021). In our systematic review, drainage output was only evaluated in the topical administration of the TXA and only in breast surgeries. All studies included in our review showed decreased rates of drainage output. Our findings are similar to Calpin et al. (Calpin et al. 2023) and opposite to Huynh et al. (Huynh et al. 2023) previous meta-analyses regarding breast surgeries.

Postoperative haematomas are one of the most significant bleeding complications of plastic surgery, as they may require a return to the hospital or operating room for drainage or evacuation (Gelidan et al. 2023). TXA has been shown to reduce haematoma formation during breast surgery (Parmeshwar et al. 2023; Weissler et al. 2020; Rifkin et al. 2023). Prevention of haematoma listed in the literature includes strict blood pressure control, early discontinuation of nonsteroidal anti-inflammatory drugs and aspirin, intraoperative hemostatic agents, postoperative pain and nausea management, and monitoring of dressing and drains (Bloom et al. 2021; Chen et al. 2023; Cason et al. 2021; Stewart et al. 2024). Our meta-analysis proves that TXA may act as a haemostatic agent to reduce haematoma formation, although our findings considered only topical administration of tranexamic acid. The total haematoma rate in patients receiving TXA was 1.2% compared to 4.3% in placebo patients.

This review recommends TXA as an adjunct for hemostasis during aesthetic surgery. However, there are no standardised recommendations for TXA in aesthetic surgery. Similar to our studies, Parmeshwar et al. (Parmeshwar et al. 2023) showed a significant reduction in haematoma formation seen in patients who received TXA in any form.

None of the included RCTs reported increased perioperative thromboembolic complications with TXA use. However, many studies excluded patients with fibrinolytic or haematologic disorders, and studies on this population are still needed. On the other hand, Masouros et al. (Masouros et al. 2022) showed that a dose of TXA 15 mg/kg is not associated with an increased risk of thromboembolic events in hip fractures. Also, Porter et al. (Porter et al. 2021) showed that the dose of 2 g of TXA, 1 g before surgery, and 1 g before wound closure was not associated with an increased risk of thromboembolic events in patients undergoing total hip replacement for hip fracture. Therefore, studies regarding appropriate doses in plastic surgery and evaluating the risk of thromboembolic events are urgently needed.

First, the main limitation of this review is that we included only 10 trials. Since few studies were included, the reliability of the results cannot be confirmed. The second limitation is the high heterogeneity of primary outcomes and the small number of included studies. Also, the subgroup analysis did not resolve the problem of heterogeneity. Finally, the surgery types and the dose of TXA differed between the studies. There were no direct comparisons of different doses among the included studies. This study’s comparison of TXA doses should be considered for research purposes. TXA is superior to placebo in reducing blood loss. There is much heterogeneity in TXA dosing strategies in the literature and few direct comparisons regarding the effectiveness and outcomes of the dosing strategy. Well-designed randomised controlled trials are still needed to evaluate the optimal dose of TXA in patients undergoing aesthetic plastic surgeries.

## Conclusions

This systematic review and meta-analysis suggest that TXA may significantly reduce total blood loss, drainage output, and haematoma formation in aesthetic plastic surgery. However, high-quality prospective studies are required to evaluate the utility of topical and intravenous TXA in these surgeries.

## Data Availability

The datasets used and/or analysed during the current study are available from the corresponding author upon reasonable request.
